# Does Top Management Team Media Exposure Affect Corporate Social Responsibility?

**DOI:** 10.3389/fpsyg.2022.827346

**Published:** 2022-02-24

**Authors:** Yichi Jiang, Liyuan Zhang, Heather Tarbert

**Affiliations:** ^1^School of Economics and Business Administration, Chongqing University, Chongqing, China; ^2^School of Business and Creative Industries, University of the West of Scotland, Paisley, United Kingdom

**Keywords:** upper echelons theory, top management teams (TMTs), media exposure, corporate social responsibility (CSR), power, political connections

## Abstract

This study examines the impact of top management team (TMT) media exposure on corporate social responsibility (CSR) and the moderating effect of TMT characteristics based on the upper echelons theory and stakeholder theory. Based on the observations of 5,352 firms between 2010 and 2019, multiple regression analysis is conducted to empirically test whether TMT media exposure can promote CSR. TMT media exposure is further divided into paper media and online media to reveal the impact of different types of TMT media exposure on CSR. Some robustness tests are also conducted to strengthen the regression results. The results found that a high level of TMT media exposure promotes social responsibility. In addition, the TMT power and political connections negatively moderate the relationship between TMT media exposure and CSR. The main contribution of this study is to develop a TMT media exposure model to assess the impact of TMT media exposure on CSR, providing a theoretical contribution to the existing literature and enriching the research in the CSR context from the perspective of the TMT characteristic moderating role.

## Introduction

In recent years, corporate social responsibility (CSR) disclosure in China has attracted increasing attention (Yin and Quazi, 2018). According to the CSR rating report of Rankings Global (RKS) in 2019, the Chinese A-share companies listed in the CSR information disclosure report increased by 129% from 2009 to 2018, with an average of 48 new companies each year. By 2000, the number of Chinese A-share listed companies increased to 134%. The growth rate of CSR report information disclosure is slightly lower than the market scale growth rate. In view of the increasingly important position of social responsibility investment in corporate operation and management and the importance of CSR for corporate sustainable development, understanding the driving of CSR has become an important research field in corporate finance literature.

According to upper echelons theory (Hambrick and Mason, 1984), corporate strategic decision-making is influenced by the characteristics of top management teams (TMTs); therefore, CSR should be regarded as a type of strategic decision which can also be decided by TMTs. Scholars have studied demographic variables from the perspective of internal control, such as gender, age, and educational background (Sánchez et al., 2017). However, Tao et al. (2019) found that external supervision encourages executives to generate better commitment in the decision-making process, while the internal characteristics of TMTs cannot effectively restrain the TMT power. Research on the external governance of executives mainly focused on government, law, and audit institutions (Allen et al., 2005). However, due to the rapid growth of China’s economy, the laws have lagged behind the development of financial markets, and more external forces are needed to supervise the company’s TMTs (Zheng, 2007). According to Atanassov and Kim (2009), extra-legal institutions, such as the media, played a critical role in influencing corporate decision-making. El Ghoul et al. (2016) also observed that media attention is an important driving force for CSR because it could influence the strength of CSR and encourage enterprises to voluntarily disclose their CSR information. Significant media attention to companies often means that companies perform in social responsibility.

Hambrick and Mason (1984) stated that the behavioural integration of TMTs comprises three main variables: the individual chief executive officer (CEO) level, the top management level, and the firm level. Previous research on media exposure and CSR has mostly focused on the firm level and has found that there is a positive correlation between media exposure and CSR (Garcia-Sanchez et al., 2014). However, most previous studies often ignore the media attention to the TMTs. Media is often the first to note the irresponsible behaviour of TMTs and protect investors by constraining managers (Zhang, 2011). It can affect corporate behaviour and promote the sustained growth of corporate value and capital markets. Media is perceived as the main source of legitimate information for investors, which helps to reduce information asymmetry for many stakeholders (Siegel and Vitaliano, 2007). Nevertheless, this process and its results are rarely examined from the perspective of the media exposure of TMTs in the CSR context. A few similar studies mainly focused on CEOs (Godos-Díez et al., 2020). Therefore, this study aims to fill this literature gap by examining a rarely considered exposure variable, namely, the media exposure of TMTs, which is determined by the frequency of their appearance in print and online.

As an important incentive mechanism, media attention is an effective substitute for the inadequacy of judicial protection and other systems in emerging markets. The media plays an intermediary role in the capital market through information communication. Even in developed western countries with relatively stringent legal and regulatory systems, the media still plays an important role in corporate governance. At the same time, with the separation of corporate ownership and control, how to introduce a more effective external governance mechanism to check and balance management teams’ self-interest behaviour and reduce agency cost has become a topic of ongoing concern amongst Chinese academics. In the process of CSR decision-making, is the individual behaviour of TMT significantly affected by the attention of external media? Is this issue still relevant in different TMT characteristics? If so, what are the differences in the individual behaviour of TMTs?

This study makes an empirical analysis of Chinese listed companies from 2010 to 2019 and uses the newspaper and online media coverage of their TMTs as a proxy to test the impact of this media exposure on their CSR. With increased media exposure, executives’ decision-making becomes more complex and diversified because they feel that their actions are being closely scrutinised and that they must consider the goals of the stakeholder groups in the decision-making process. The relationship between TMT media exposure and CSR is also expected to be affected by TMT characteristics. This article further discusses the moderating effects of TMT power and political connections.

The main contributions of this research are as follows. Firstly, as the world’s second-largest economy and largest emerging capital market, it is an important supplement to study the impact of TMT media exposure on CSR in China. From the perspective of media attention to TMTs, this study finds that an increase in such attention may enhance the strength of TMT voices in their companies, improve the effectiveness of external supervision, and have a positive impact on CSR. It demonstrates that the media news exposure of TMTs in emerging economies can play a monitoring and governance role. Secondly, the existing literature on CSR tends to focus on the economic consequences of firm-level media coverage but has not examined the impact of TMT-level media coverage. This study examines CSR practices by investigating the media exposure to TMTs so as to provide a basis for the follow-up research of the relationship between TMT media coverage and other corporate behaviours. Finally, this study investigates the moderating effects of TMT power and political connections, and reveals their inhibition of TMT media exposure on CSR governance.

The remainder of this article is structured as follows. First, the literature review and hypotheses tested are presented, followed by the study’s sample, methodology, and results. Finally, the discussions and proposals for possible areas of future research development are discussed.

## Literature Review and Hypotheses

### Literature Review

#### The Consequences of Media Exposure

Most scholars hold that the media, as an informal governance mechanism, plays a positive role in corporate behaviour (Lim et al., 2017). This continuous overview can promote the function of informing stakeholders while alleviating the adverse effect of information asymmetry (Bushee et al., 2010) in addition to reducing the motivation for, and the possibility of, illegal management. Dyck et al. (2008) studied the media’s governance function and found that it played a governance role through mechanisms of reputation and legal constraints. In light of China’s underdeveloped manager market and imperfect reputation mechanism, Huang and Li (2015) pointed out that the Chinese media mainly achieves governance through administrative intervention. In terms of the tone of media reports, Bednar et al. (2013) suggested that negative news report of a firm is related to subsequent strategic changes. In contrast, positive reports on a CEO can lead to overconfidence and increase corporate risk-taking (Chatterjee and Hambrick, 2011).

In addition to media exposure at the firm level, as the policymakers and executors of enterprises, media exposure of TMTs also influences corporate business behaviours (Górska and Mazurek, 2021). The CEO’s image may indirectly affect the image of the enterprise products (Alghawi et al., 2014). Thus, the media coverage of a CEO can be regarded as a marketing tool. Such media coverage can also increase investors’ understanding of the company, promote the reputation of the CEO and the company, and attract more investors to buy the company’s shares (Carter, 2006). Kang and Han Kim (2017) found that CEOs can improve their compensation through media coverage, which, in turn, affects the company’s performance. Some scholars also directly equated CEO exposure with CEO reputation (Weng and Chen, 2017). Media exposure of CEOs can enhance firm-specific information into stock prices, reducing stock price synchronicity (Li et al., 2019).

#### Influence of Corporate Social Responsibility

The existing analysis of the driving factors of CSR is mainly divided into the research on TMT characteristics, internal governance structure, and external public pressure. Firstly, the TMT characteristics can affect the fulfilment of CSR. Galbreath (2011) found that some characteristics of female executives, such as lack of self-confidence, kindness, and thoughtfulness, made it easier for them to adopt CSR policies. Wen and Song (2017) also believed that managers who received overseas cultural education could better fulfil CSR. Secondly, internal governance structure affects the participation of CSR. Wang et al. (2020) found that the ownership structure of multiple major shareholders is an effective internal governance mechanism. Specifically, the ownership structure of multiple large shareholders could restrict the controlling shareholders from infringing the interests of minority shareholders, thus promoting social responsibility. From the perspective of board structure, Liao et al. (2018) posited that the larger the board size and separation between the CEO and chairman positions and the female directors, the easier it is to promote the fulfilment of CSR. Thirdly, it is found that external public pressure can affect CSR. Gandullia and Piserà (2020) observed that effective average corporate tax reduced the level of CSR. However, effective tax policies encourage large and medium-sized enterprises to disclose information and assume social responsibility. Moreover, Zheng et al. (2014) found that under the supervision pressure of external governments, state-owned enterprises are more inclined to disclose CSR in detail.

Brown and Deegan (1998) made a preliminary contribution to the study of the impact of corporate media coverage on CSR. Specifically, based on the media agenda setting theory and the legitimacy theory, they explained the annual report disclosure of Australian companies in the first study, using media coverage as a proxy of social concern, and concluded that variations in media attention have a positive correlation with variations in management information disclosure. Thereafter, Lindgreen et al. (2009) found that the higher the levels of media attention given to listed companies, the more list companies pay attention to their external corporate image and the more they paid attention to environmental protection and other aspects of social responsibility investment. At the same time, more CSR information would be disclosed voluntarily. Subsequently, according to the legitimacy theory, Islam and Deegan (2010) conducted the first study on how developing country companies responded to media attention, emphasising that media supervision, as an external supervisory force, plays an important role in motivating companies to actively take responsibility for their stakeholders. Reverte (2009) demonstrated that as an informal system and independent supervisor of third parties, media attention is one of the important deciding factors in corporate decisions to voluntarily disclose CSR information, while also being an important driving force for them to fulfil their social responsibilities. In summary, media attention significantly improves the willingness and level of voluntary social responsibility information disclosure (Li et al., 2017).

To summarise, most scholars supported the idea that the media pays attention to the role of supervision and governance. However, most of the media exposure research focuses on the analysis of the firm level and seldom studies the role of media governance in TMTs. Up to now, there is little research on the supervisory role of media exposure of TMTs.

### Hypothesis Development

According to the stakeholder theory, as an important external stakeholder of enterprises, the news media and their reports on executives have an important impact on managers’ decision-making and behaviour (Delmas, 2001). When the media pays close attention to executives, this exposure can form a “spotlight effect,” that is, the management reported upon becomes the focus of public opinion (Qi et al., 2014), thereby causing the public and investors to have higher expectations for enterprises to fulfil CSR expectations. At the same time, upper echelons theory posits that the effective implementation of TMT organisational strategies plays a key role, and the final organisational output is influenced by the strategic formulation and implementation. In addition, the behaviour of TMTs can directly affect CSR. Following Dyck and Zingales (2002) research, this section highlights three ways in which media coverage of TMTs affects their CSR performance.

Firstly, there is a reputation constraint mechanism (Dyck et al., 2008). Many researchers regard the level of CEOs’ media exposure as an alternative variable of CEO reputation (Francis et al., 2008). TMTs realised that their appearances in the media had affected their image and reputation (Love et al., 2017). Extensive media attention to TMTs can also improve managers’ transparency. Considering the company’s long-term future interests, the possibility of TMTs seeking private interests becomes very low. Thus, they tend to actively disclose CSR information in order to obtain the support of investors and other stakeholders and maintain a positive image (Lindgreen et al., 2009).

The second way is the external market pressure, such as government agencies, the public, and the media or stakeholders. Xiong and Luo (2021) found that public pressure, such as media attention, may force TMTs to reduce opportunistic behaviour and corporate risk and improve corporate value. At the same time, stakeholders are also concerned about media reports, which may bring greater environmental pressure to the company.

The final way is administrative intervention (Huang and Li, 2015). Media attention to TMTs can increase the pressure of potential government intervention, make them vulnerable to intervention by administrative bodies, and increase the likelihood to be punished by the administrative authorities. In order to meet the needs of sustainable development, TMTs are often more willing to voluntarily fulfil their social responsibility. Yang and Zhao (2012) documented that only when the government and administrative departments participate in media supervision can it play a role in governance. At the same time, TMTs are facing huge reputation loss and may also be subject to administrative punishment due to constant media exposure. Thus, the best countermeasure for TMTs receiving extensive media attention is to consciously fulfil their CSR requirements.

In short, companies whose TMTs are highly exposed by the media are faced with a more complicated stakeholder environment, greater expectations and pressure of stakeholders, and more social responsibilities to be fulfilled. As the disseminator of information, media exposure helps to reduce the information asymmetry of the market. The relatively transparent environment enables stakeholders to understand any potential “crisis” in TMT operations, which makes it difficult for these teams to take actions that harm the public interests (Chen et al., 2013). Based on the above analysis, the following hypothesis is proposed.

H1: The level of TMT media exposure can promote the better fulfilment of CSR.

Haynes and Hillman (2010) believed that power plays an important role in strategic decision-making and strategic differences. TMT power (Finkelstein, 1992) refers to the power that TMTs have to continuously influence the key corporate decisions despite the potential opposition of other directors. Based on the upper echelons theory, when TMTs are powerful, their personal preferences will be well reflected in strategy making (Haynes and Hillman, 2010). Numerous studies have shown views that TMT power is negatively related to CSR (Rashid et al., 2020). Based on the managerial opportunism hypothesis, powerful executives are more confident in their business decisions to pursue the personal reputation and compensation increase brought about by the expansion of the company, which leads to blind over-investment and damage to the stakeholders. On the contrary, the weak TMTs in power are more worried about their own corporate decisions, considering taking on more social responsibilities, easing the relationship between the company and its stakeholders, and thus stabilising their careers.

The topic applied to Hypothesis 2 is that powerful executives are not interested in disclosing CSR. Instead, they prefer to invest in other profitable activities (Rashid et al., 2020). At the same time, when TMTs are with too much power, the media or shareholders’ supervision of management is reduced (Jensen and Meckling, 1976), which leads to the weakening of supervision motivation and lack of supervision information. This, therefore, makes executives override corporate governance, have important control rights over the company, and formulate self-interest policies under the temptation of rent-seeking. In other words, powerful TMTs play a role of a “protective cover” for media exposure which reduces the sensitivity of enterprises to the environment. According to agency theory, with the increase of TMT power, under the lack of effective supervision, TMTs are more likely to ignore the corporate interests and seek benefit for themselves. In addition, in the process of strategy-making, powerful TMTs can significantly weaken the influence of industry standards on executive strategy-making, thus making it easier for enterprises to make strategic decisions deviating from industry standards (Tang et al., 2011). As a result, TMT power is too large or even above external supervision, and it is more likely to be “rent-seeking.” In short, TMT media exposure enhances CSR, but when TMTs have too much power, the relationship between the two variables is weakened. Therefore, the positive correlation between TMT media exposure and CSR may be disturbed by TMT power.

H2: TMT power negatively moderates the impact of TMT media exposure on CSR.

Regarding political connections, as Allen et al. (2005) state in their article, political connections refer to the implicit political relationship between the company and individuals with political rights. In developing countries and regions, firms tend to get policy support and resource allocation through informal alternative mechanisms, and political connections can help firms eliminate the obstacles caused by institutional weaknesses.

Based on the upper echelons theory, when TMTs have political connections, they use these political resources to exert influence on the media and ensure that media reports produce “selective bias” or avoid important points so as to maintain their own reputation, thus weakening the media’s governance role. TMTs with political connections play the role of “amulets.” Due to the lack of effective supervision, politically connected companies are discouraged from improving the quality of their financial information disclosure (Chaney et al., 2011) to the company’s violations, and the self-interest behaviours of its managers cannot be effectively reported by the media, significantly reducing the willingness of companies to fulfil their CSR. In this sense, political connections play a counter-role in improving capital market efficiency. Due to the lack of an invisible “shelter” for companies without political connections, the degree and content of company information disclosure are greater, allowing investors to better understand corporate performance. Wang et al. (2018) found that compared with politically affiliated enterprises, the media plays a greater role in monitoring the private interests of the controlling shareholders of non-politically affiliated enterprises. Therefore, media attention has a stronger driving force to CSR disclosure of listed companies without political connections. Consequently, we concluded that political ties tend to inhibit the supervision and governance of the media. This leads to the third hypothesis.

H3: TMT politically connected firms negatively moderate the impact of TMT media exposure on CSR.

Based on Hypotheses 1, 2, and 3, the conceptual framework of this article is shown in the figure below.


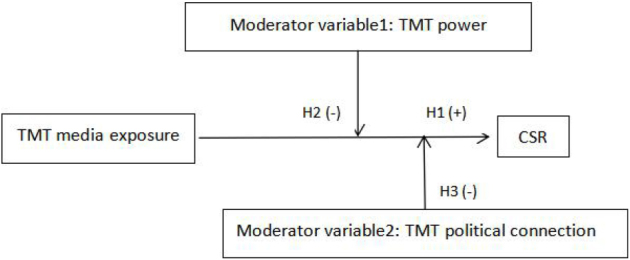


## Study Design

### Date and Samples

Listed companies that issued CSR reports in the Shanghai and Shenzhen Stock Exchanges from 2010 to 2019 are selected as the research sample. To ensure the quality of the data, we excluded financial, ST, ST*, and PT listed companies, companies with missing data and no CEO, and companies with less than 1 year of operation during the survey period. Therefore, we finally obtained 5,352 firm-year observations. The CSR data are derived from RKS’s CSR Ratings^1^, including the executive news data, TMT power data, political connections data, and other financial information that were extracted from the China research data service platform, CNRDS. Other control variable data are extracted from the CAMAR database. To avoid the influence of outliers on the regression results, all variables are winsorised at the tail of the upper and lower 1% distribution.

### Measures

#### Dependent Variable

Based on the articles of Carroll (1999) and Dahlsrud (2008), and in combination with the development status of CSR, this current study defines CSR as a corporate social responsibility where an enterprise undertakes economic responsibility to shareholders (pursuing shareholders’ interests), in which it also needs to pay attention to employees, creditors, customers, suppliers, government, and responsibilities of other stakeholders such as the community and the ecological environment.

The CSR reports rating results of listed companies released by RKS’s CSR Ratings (Rezaee et al., 2020) are used to measure CSR in this article. As the CSR rating report of RKS has four characteristics of the macrocosm (M score), content (C score), technique (T score), and industry (I score), the score is comprehensive, fair, and reliable. The CSR score referred to in this study is the total comprehensive score of MCTI, which indicates the corporate CSR performance in the current year, with a full score of 100. The higher the social responsibility score, the more social responsibility the enterprise undertakes. Hence, a better CSR performance.

#### Independent Variable

There are many views on the definition of top management teams (TMTs) in academia (Carpenter et al., 2004), and different scholars have different understandings. Hambrick and Mason (1984) defined the TMTs as all senior managers while Finkelstein and Hambrick (1990) defined them as board members and senior managers. Referring to the definition in Hambrick and Mason (1984) and based on the actual situation in China and the availability of data, this article defines the TMTs as all the senior managers of the management level above the deputy general manager of the company, including the president, vice president, general manager, deputy general manager, general manager assistant, chief accountant chief engineer, and other senior managers of the management level disclosed in the annual report of the listed company.

Referring to Khan and Sukhotu (2020), this article defines media exposure as the number of times observations appear in both print and electric media. Since few people have studied the media exposure on TMTs, this article refers to the definition of CEO media exposure in Godos-Díez et al. (2020) and Wang et al. (2020) combined with the actual situation of China and the availability of data, TMTs are defined as the number of times TMTs appear on the print and internet media. The natural logarithm of “1++total TMT internet media coverage+total TMT press media coverage” is used to measure the overall media exposure of the TMTs (Media_total), while the natural logarithm of “1+total TMT internet media coverage” is used to measure the exposure of the TMTs in emerging online media (Media_net). The natural logarithm of “1+total TMT press media coverage” is used to measure the media exposure of the TMTs in traditional newspapers (Media_paper). The total media exposure included positive, neutral, and negative reports. Referring to Ning et al. (2020), all the media coverage of the TMTs is obtained from the China Executive News Database (CEND).

#### Moderating Variables

Many researchers have proposed that the CEO pay slice can be used to measure CEO power. The most authoritative representative is Bebchuk et al. (2011). According to this view, “the CEO Pay Slice is defined as the percentage of the total compensation to the top five executives that go to the CEO.” Based on the actual situation in China and the availability of data (only the data of the top three executives’ compensation can be obtained), this article uses the following way to measure TMT power. TMT power is measured by the top three executives’ compensation divided by the total compensation of all executives.

The second moderating variable is TMT political connections. Referring to Fan et al. (2007) and Wang et al. (2017), the definition of TMT political connection in this article is that the TMT political connection of a company depends on whether any TMT is or has been a provincial, municipal, or local government official. We manually collect the annual report of the enterprise and check the resumes of the company’s TMTs to determine whether any TMTs are or have been provincial, municipal, local government officials, deputies to the National People’s Congress, and/or members of the National Committee of the Chinese people’s Political Consultative Conference. This article measures the degree of political relevance of a company’s TMT by the proportion of TMTs with political relevance among TMT members.

To control the influence of other factors, this article selects the following two types of control variables with reference to Darmawan et al. (2019) research. The first category is the corporate level characteristic control variables. This article selects firm size (SIZE), asset-liability ratio (LEV) (Aharon and Yagil, 2019), return on assets (ROA) (Bollazzi and Risalvato, 2018), product market competition (HHI), shareholding ratio of the largest shareholder (Top1) (Peng and Yang, 2014), nature of property right (State), and firm age (Firm_age). The second category is the TMT level characteristic control variables. This article selects the proportion of females in TMTs (Female_ratio), TMT age (Ave_age), TMT educational level (Ave_edu), Board size (Boardsize), and duality (Duality). The specific definition and calculation methods of all variables are shown in Table 1.

**TABLE 1:** Variable definitions and measurement.

Variable type	Variable name	Variable Symbol	Variable definition
Dependent variable	Corporate Social Responsibility	CSR	CSR Ratings score of listed companies on RKS.
Independent variable	TMTs media exposure	Media_total	The natural logarithm of “1+total internet media coverage+total press coverage”
		Media_net	The natural logarithm of “1+total internet media coverage”
		Media_paper	The natural logarithm of “1+total press media coverage”
Moderating variables	TMTs Power	Power	Total compensation of top three executives divided by total compensation of all executives
	TMTs Political	Political	TMTs political connection, measured by the proportion of TMTs with political relevance among TMT members.
Control variables	Proportion of female	Female_ratio	The proportion of female TMT
	Ave_age	Ave_age	The average age of TMT
	TMTs education level	Ave_edu	A categorical variable of TMTs education level
	Board size	Boardsize	Total number of directors
	TMTs shareholding ratio	Gm_holding	Measured by the ratio of total TMTs holdings to firm’s total shares outstanding
	Duality	Dual	A dummy variable that takes 1 if the CEO is also the chairman of the board and 0 otherwise
	Firm Size	Size	Size of a firm, measured by the natural logarithm of total assets
	Financial leverage	Lev	Financial leverage of a firm
	Corporate performance	Roa	Return on the enterprise’s total assets
	Product market competition	HHI	Indicator of the industry concentration, using the Herfindahl-Hirschman index (HHI)
	Ownership concentration	Top1	The concentration ratio of shares is expressed by the proportion held by the largest shareholder in the current year
	State	State	The property nature of state-owned enterprises (State) is 1, otherwise it is 0.
	Firm_age	Firm_age	The age of firm

### Empirical Model

To test the impact of TMT media exposure on CSR, we constructed the following baseline empirical model. Based on the above analysis, if Hypothesis 1 is true, then α_1_ would be significantly greater than 0.


(1) $$CSR_{i,t}=\alpha_{0}+\alpha_{1}Media_{i,t}+\sum a_{j}Controls_{i,t}+Year+Ind+\varepsilon_{i,t}$$

In order to test Hypotheses 2 and 3, based on the model (1), this article adds TMT power (Power), TMT political (Political), and their interaction term with TMT media exposure (Media × Power and Media × Political) to test the moderating effect of TMT characteristics on the impact of TMT exposure and CSR, which is for model (2) and model (3) respectively.


(2) $$CSR_{i,t}=\beta_{0}+\beta_{1}Media_{i,t}+\beta_{2}Power_{i,t}+\beta_{3}Media_{i,t^{*}}Power_{i,t}+\sum \beta_{j}Controls_{i,t}+Year+Ind+\varepsilon_{i,t}$$

(3) $$CSR_{i,t}=\chi 0+\chi 1Media_{i,t}+\chi 2Political_{i,t}+\chi 3Media_{i,t^{*}}Political_{i,t}+\sum \chi_{j}Controls_{i,t}+Year+Ind+\varepsilon_{i,t}$$

## Results

### Descriptive Statistics

This article makes descriptive statistics on 5,352 firm-year observations. Table 2 shows the descriptive statistics of the variables. For the explained variable (CSR), the mean value of score is 40.13, the standard deviation is 12.36, the minimum score is 17.24, and the maximum score is 84.13, indicating that there are still big differences in CSR information disclosure between the listed companies in China. Overall, the CSR performance of China’s listed companies remained low, and there exists much room for improvement. For explanatory variables (Media_total), the mean value of total coverage News is 3.151, the standard deviation is 1.518, the minimum value is 0.693, and the maximum value is 7.643, showing that there are great differences in the financial media exposure of different executives of Chinese listed companies. When comparing online news coverage and newspaper news coverage, it is found that the mean value, standard deviation, and maximum value of News_online are higher than those of News_paper, revealing that with the technological progress, the influence of the Internet as a medium of financial information dissemination which has surpassed that of traditional newspapers.

**TABLE 2:** Descriptive statistics.

Variables	Observations	Mean	SD	Minimum	Median	Maximum
CSR Score	5,352	40.13	12.36	17.24	37.57	84.13
Media_total	5,352	3.151	1.518	0.693	2.944	7.643
Media_online	5,352	2.927	1.565	0	2.773	7.560
Media_paper	5,352	1.515	1.380	0	1.386	5.700
Power	5,352	0.998	0.282	0.282	0.964	2.388
Political	5,352	0.048	0.057	0	0.040	0.278
Female_ratio	5,352	0.158	0.101	0	0.143	0.471
Ave_age	5,352	50.18	3.086	37.88	50.18	58.64
Ave_edu	5,352	3.478	0.477	2	3.522	5
Boardsize	5,352	9.210	1.961	5	9	15
Gm_holding	5,352	0.029	0.089	0	0	0.637
Dual	5,352	0.183	0.387	0	0	1
Size	5,352	23.27	1.477	19.58	23.13	27.42
Lev	5,352	0.495	0.202	0.038	0.510	0.973
Roa	5,352	0.076	0.131	−2.543	0.080	0.410
HHI	5,352	0.127	0.148	0.016	0.077	1
Top1	5,352	0.367	0.161	0.080	0.351	0.758
State	5,352	0.565	0.496	0	1	1
Firm_age	5,352	18.76	5.788	1	18	53

Table 3 shows the Pearson correlation coefficient matrix among the key variables. The correlation coefficients among Media_total, Media_online, Media_paper, and the Score are 0.230, 0.222, and 0.217, respectively, and all of them have passed the 1% significance level test. It is preliminary verification that more TMT media exposure is associated with better CSR performance. The correlation coefficients of the two moderating variables indicate that TMT power and TMT political connection hinder the relationship between TMT media exposure and CSR. Hypothesis 1 is initially supported. The correlation coefficient matrix can only be used as a very preliminary analysis, and multiple regression analysis and statistical analysis are required to gain insight into the real relationships between the variables.

**TABLE 3:** Correlation matrix.

	CSR Score	Media_total	Media_online	Media_paper	Power	Political
CSR Score	1					
Media_total	0.230***	1				
Media_online	0.222***	0.979***	1			
Media_paper	0.217***	0.835***	0.737***	1		
Power	−0.112***	−0.073***	−0.060***	−0.089***	1	
Political	−0.014*	0.144***	0.129***	0.180***	0.047***	1

* and *** Denote statistical significance at the 10 and 1% levels, respectively.

### Multiple Regression Analysis

#### Top Management Team Media Exposure and Corporate Social Responsibility

Table 4 shows the influence of TMT media exposure (Media_total) on the CSR Score of listed companies. There is a significant positive regression coefficient (α = 0.785, *p* < 0.001) which demonstrated that the more TMT media exposure (Media_total), the better CSR practice. That is, the strong pressure from public opinion and the public supervision of media exposure are beneficial to the improvement of CSR performance. This finding supports the theoretical expectations of Hypothesis 1. The results of online news and newspaper news also pass the significance test, and the regression coefficient is positive.

**TABLE 4:** Multiple regression analysis.

Variables	(1)	(2)	(3)	(4)	(5)	(6)
	CSR Score	CSR Score	CSR Score	CSR Score	CSR Score	CSR Score
Media_total	0.785***			0.729***		
	(7.11)			(6.71)		
Media_online		0.728***			0.682***	
		(6.87)			(6.52)	
Media_paper			0.857***			0.785***
			(6.76)			(6.27)
Size	3.664***	3.703***	3.667***	3.269***	3.298***	3.288***
	(23.42)	(23.81)	(23.21)	(20.33)	(20.56)	(20.40)
Lev	−5.373***	−5.391***	−5.491***	−4.948***	−4.963***	−5.051***
	(−5.63)	(−5.65)	(−5.75)	(−5.29)	(−5.31)	(−5.40)
Roa	1.689*	1.775*	1.373	2.016**	2.095**	1.740*
	(1.72)	(1.81)	(1.39)	(2.11)	(2.20)	(1.82)
HHI	6.710***	6.715***	6.470***	6.285***	6.289***	6.065***
	(2.97)	(2.97)	(2.86)	(2.82)	(2.82)	(2.72)
Top1	2.829***	2.788***	2.687**	2.891***	2.860***	2.746***
	(2.70)	(2.66)	(2.57)	(2.81)	(2.77)	(2.67)
State	0.971***	0.962***	0.870***	0.112	0.096	0.050
	(2.93)	(2.90)	(2.64)	(0.31)	(0.27)	(0.14)
Firm_age	0.080***	0.079***	0.080***	0.071***	0.070**	0.072***
	(2.94)	(2.87)	(2.93)	(2.59)	(2.52)	(2.61)
Female_ratio				2.805*	2.772*	2.810*
				(1.81)	(1.79)	(1.82)
Ave_age				0.226***	0.230***	0.210***
				(4.02)	(4.10)	(3.72)
Ave_edu				2.445***	2.429***	2.527***
				(8.06)	(7.99)	(8.36)
Boardsize				0.498***	0.504***	0.483***
				(5.81)	(5.87)	(5.66)
Gm_holding				6.195***	6.135***	6.587***
				(4.16)	(4.12)	(4.41)
Dual				−1.563***	−1.568***	−1.552***
				(−4.31)	(−4.32)	(−4.28)
_cons	−55.302***	−55.622***	−54.536***	−70.107***	−70.486***	−69.081***
	(−15.38)	(−15.45)	(−15.04)	(−16.82)	(−16.92)	(−16.35)
Industry/Year	Yes	Yes	Yes	Yes	Yes	Yes
N	5,352	5,352	5,352	5,352	5,352	5,352
adj. *R*^2^	0.362	0.361	0.361	0.378	0.378	0.377

*, **, and *** Denote statistical significance at the 10, 5, and 1% levels, respectively. The t values are in parentheses.

In terms of control variables, firm size has a significant positive regression coefficient which indicates that the larger the company size, the better its CSR performance, which is similar to the conclusions of Wickert et al. (2016). There is a significant negative correlation between financial leverage and CSR. Particularly, the significance level stands at 1%, suggesting that the better a company’s financial situation is, the higher its CSR performance. At the same time, profitability has improved CSR performance at the 1% significance level. Finally, compared with non-state-owned enterprises, the CSR performance of state-owned enterprises is significantly higher.

The results of heterogeneity estimation are reported in Table 4. From column (1) to column (3), corporate-level characteristic control variables are added. The regression coefficients are 0.785, 0.728, and 0.857, respectively, and passed the significance level test of 1%. From columns (4) to columns (6), control variables at the TMT level characteristic variables are further added. The regression coefficients of A, B, and C are 0.729, 0.682, and 0.785, respectively, and all passed the significance level test of 1%.

In summary, as can be seen from the regression results in Table 4, regardless of the use of Media_total, Media_online, or Media_paper as an explanatory variable and no matter what control variables are considered, there is a significant positive correlation between TMT media exposure and CSR. Hence, Hypothesis 1 of this article cannot be rejected. We, therefore, find that TMT power negatively moderates the impact of TMT media exposure on CSR according to our results.

#### Moderating Variable Regression Results

The moderating variable TMT power and its interaction term (Media_total × Power, Media_online × Power, and Media_paper × Power) are further introduced into the observations to investigate the moderating effect of TMT power. The regression results are shown in Table 5. To observe whether the impact of TMT media exposure on CSR differs due to the heterogeneity of executive team characteristics, TMT power is added as the first moderating variable in this article.

**TABLE 5:** Top management team (TMT) media exposure and corporate social responsibility (CSR): the impact of TMT power.

Variables	(1)	(2)	(3)
	Score	Score	Score
Media_total	1.408***		
	(4.22)		
Power	0.053	0.138	−1.322***
	(0.05)	(0.14)	(−1.89)
Media_total × Power	−0.688*		
	(−2.18)		
Media_net		1.451***	
		(4.46)	
Media_net × Power		−0.766**	
		(−2.50)	
Media_paper			1.272***
			(3.37)
Media_paper × Power			−0.512
			(−1.42)
Controls	Yes	Yes	Yes
Industry/Year	Yes	Yes	Yes
N	5,352	5,352	5,352
adj. *R*^2^	0.391	0.391	0.390

*, **, and *** Denote statistical significance at the 10, 5, and 1% levels, respectively. The t values are in parentheses.

In Table 5, we can find that the regression coefficient between the interaction term (Media_total × Power) and CSR (Score) is −0.688, which is significantly negative at the level of 10%. It shows that the positive correlation between TMT media exposure and CSR may be interfered by TMT power. When the independent variables are Media_net and Media_paper, the results are basically the same. The regression results thereby support research Hypothesis 2.

We further examine the influence of TMT political connections (another important TMT characteristic) on TMT media exposure and CSR. In Table 6, we can find that the regression coefficients between interactive items (Media_total × Political, Media_online × Political, and Media_paper × Political) and CSR (Score) are −3.116, −3.001, and −2.499, respectively, all of which are significantly negative at least at the level of 10%, which shows that when the degree of TMT political connection is relatively low, the positive impact of TMT media exposure on CSR is stronger. The regression results support the research of Hypothesis 3.

**TABLE 6:** TMT media exposure and CSR: the impact of TMT political connection.

Variables	(1)	(2)	(3)
	Score	Score	Score
Media_total	0.040		
	(0.43)		
Political	12.086**	10.920**	6.212
	(2.46)	(2.40)	(1.62)
Media_total × Political	−3.116***		
	(−2.74)		
Media_online		0.079	
		(0.89)	
Media_online × Political		−3.001***	
		(−2.79)	
Media_paper			−0.074
			(−0.65)
Media_paper × Political			−2.499*
			(−1.95)
Controls	Yes	Yes	Yes
Industry/Year	Yes	Yes	Yes
N	5,352	5,352	5,352
adj. *R*^2^	0.342	0.342	0.342

*, **, and *** Denote statistical significance at the 10, 5, and 1% levels, respectively. The t values are in parentheses.

### Robustness Tests

We explore the impact of possible reverse causality on the research conclusions. The topic discussed in this article is that more TMT media exposure promotes CSR performance. At the same time, those companies with better CSR performance will receive more media exposure. Therefore, the research result of this article may be affected by reverse causality. Therefore, in order to solve this problem, this article refers to Zuo et al. (2020) in using the per capita postal and telecommunications traffic (Ave_traffic) to represent the information level and using it as Instrumental Variable for generalised moment estimation (GMM). GMM is measured by the sum of the total postal services plus the total telecommunications services and then divided by the total population in the region. Generally speaking, the higher the informatisation level of the region where the listed companies are located, the more information the media will expose on the listed company under the market competition. Consequently, the development level of regional postal and telecommunications services will not directly affect the CSR decision-making of the listed company. Therefore, the selected tool variables meet the requirements of “correlation” and “exogenous.” The first and second columns of GMM in Table 7 report the respective regression results of instrumental variables. The first column is the regression results of the first stage, which shows that the significance of Ave_traffic and Media_total is positive at the level of 5%. The second column in GMM is the second stage regression result, which shows that the significance of Media_total and CSR Score is positive at the level of 1%. This further shows that the empirical results of this article are not disturbed by reverse causality.

**TABLE 7:** Robustness tests.

Variables	GMM		Fixed effect model			Redefining CSR		
	Media_total	Score	Score	Score	Score	Scvps	Scvps	Scvps
Media_total		6.461***	2.216***	0.279	0.813***	0.065***	0.063*	0.086***
		(11.08)	(7.43)	(1.20)	(5.74)	(4.60)	(1.79)	
Power				0.085			−0.143	
				(0.10)			(−0.52)	
Media_total × Power				−0.397*			−0.005**	
				(−1.82)			(−2.05)	
Political					11.659**			0.666
					(1.97)			(1.02)
Media_total × Political					−1.921**			−0.376*
					(−2.20)			(−1.89)
Ave_traffic	0.001**							
	(2.36)							
Controls	Yes	Yes	Yes	Yes	Yes	Yes	Yes	Yes
Industry/Year	Yes	Yes	Yes	Yes	Yes	Yes	Yes	Yes
*N*	5,352	5,352	5,352	5,352	5,352	5,352	5,352	5,352
adj. *R*^2^	0.342	0.301	0.314	0.318	0.389	0.441	0.441	0.442

*, **, and *** Denote statistical significance at the 10, 5, and 1% levels, respectively. The t values are in parentheses.

Next, we use the fixed-effects model regression analysis to examine the effect of TMT media exposure on CSR and confirmed that our results do not qualitatively differ from the findings reported in Table 7’s fixed-effect model column. We again ran fixed-effects regressions and control for unobservable characteristics that are time-invariant. The fixed-effects model regression results were consistent, suggesting that our conclusion is not vulnerable to the omitted-variable bias.

Finally, we refined the CSR measurement method to strengthen the research conclusions. The measures of the explanatory variables are replaced and the empirical results are retested. Referring to Noronha et al. (2018), the social contribution value per share (SCVPS) is used to measure CSR. SCVPS was brought up by the Shanghai Stock Exchange in 2008. The regression results are reported in Table 7, Redefining CSR column. It can be seen that the regression coefficient between TMT media exposure and SCVPS is 0.065 and is significantly positively correlated at the 1% level. The regression coefficients of the interaction terms of the moderating variables (Media_total × Power and Media_total × Political) and SCVPS are −0.005 and −0.376, respectively, showing a significant negative correlation at least at the 10% level. It can be seen that the findings of this article remain unchanged.

### Further Tests

#### Different Corporate Social Responsibility Dimensions

We test the impact of TMT media exposure on different CSR dimensions. The rating results, combined with the reality of the Chinese context, are used to classify CSR into four dimensions, namely, Macrocosm (M), Content (C), Technique (T), and Industry (I), and to evaluate the quality of CSR. Macrocosm focused on the strategic management of CSR, with a weight of 30%; Content focused on the actual performance of CSR, with a weight of 45%; Technique mainly focused on CSR information disclosure, with a weight of 15%; and Industry evaluated the industry characteristics of the enterprise, with a weight of 10%. The influence of the TMT media exposure on different CSR dimensions is tested, and the results show that the regression coefficients of executive media reports on the four dimensions of CSR are all significantly positive (Table 8). This indicates that the impact of the TMT media exposure on enhancing CSR is mainly achieved by strengthening CSR strategy formulation, promoting CSR strategy implementation, and improving CSR information disclosure.

**TABLE 8:** TMT media exposure and CSR: Different CSR Dimensions.

Variables	(1)	(2)	(3)	(4)
	M	C	T	I
Media_total	0.205***	0.323***	0.104***	0.092***
	(5.36)	(5.96)	(6.41)	(6.61)
Controls	Yes	Yes	Yes	Yes
Year/Industry	Yes	Yes	Yes	Yes
N	5,352	5,352	5,352	5,352
adj. *R*^2^	0.416	0.336	0.426	0.469

*** Denotes statistical significance at the 1% level. The t values are in parentheses.

#### Distinguish Media Exposure Tone

Given that negative media reports generally criticise some problems or improper behaviours of executives of listed companies, the supervision and dissemination of negative reports are more obvious than positive and neutral reports. Therefore, this study further distinguishes the tone of media reports into positive and negative. On the one hand, when negative events occur, the TMT with political connection and power may use political authority to influence the tendency of the media so as to reduce the governance role of the media. On the other hand, due to the lack of invisible shelter, the media can give full play to the role of supervision and governance. Therefore, the attention of the media is more binding on the earnings management behaviour of non-political and less powerful TMTs and related listed companies. Therefore, compared with the measurement of the number of media exposure, negative media reports can induce administrative intervention, hence, the impact on the TMT is more obvious.

Therefore, this article further tests and distinguishes the tone of media reports. Table 9 shows that the regression coefficient is 0.776 when the independent variable is negative media coverage, which is greater than 0.446 when the independent variable is positive media coverage. In addition, the test of the interaction term shows that it is significant only when the independent variable is negative media coverage. This result confirms the research of Islam and Deegan (2010) which stated that when TMTs have negative media reports because they are worried about the intervention of the regulatory authorities, TMTs focus on the implementation of CSR.

**TABLE 9:** Distinguish media exposure tone.

Variables	Negative News (3)			Positive News		
	(1) Score	(2) Score	(3) Score	(4) Score	(5) Score	(6) Score
Media_total	0.776***	1.173***	0.522***	0.446***	1.197***	0.868***
	(7.20)	(3.71)	(4.22)	(4.77)	(3.76)	(6.19)
Power		−0.863			−1.151	
		(−1.19)			(−1.54)	
Media_total × Power		−0.703**			−0.437	
		(−2.41)			(−1.48)	
Political			9.139**			9.829**
			(2.37)			(2.25)
Media_total × Political			−1.563**			−2.204
			(−2.10)			(−1.40)
Controls	Yes	Yes	Yes	Yes	Yes	Yes
Industry/Year	Yes	Yes	Yes	Yes	Yes	Yes
*N*	5,352	5,352	5,352	5,352	5,352	5,352
adj. *R*^2^	0.375	0.389	0.376	0.389	0.392	0.390

** and *** Denote statistical significance at the 5 and 1% levels, respectively. The t values are in parentheses.

## Conclusion

### Concluding Remarks

This study takes the listed companies in Shanghai and Shenzhen Stock Exchanges from 2010 to 2019 as its research object to examine the relationship between the level of media attention paid to their TMTs and CSR in Chinese markets. The study investigates the influence of executives’ characteristics on the media’s role of supervision and governance and information transmission, paying special attention to the differences of TMT power and political connection. The empirical results show that TMT media exposure can promote CSR. Powerful TMT and political connection inhibit the relationship between TMT media exposure and CSR.

### Theoretical Contributions and Practical Implications

#### Theoretical Contributions

Firstly, based on the upper echelons theory and stakeholder theory, combined with the characteristics of Chinese enterprises, this study focuses on how TMT media exposure affects corporate social responsibility. Since the upper echelons theory was put forward, scholars have continuously combined new concepts with the upper echelons theory. At the same time, they have responded to the call of Hambrick and Mason (1984) to “Introduce new variables into the upper echelons theory.”

However, most of the researchers base their discussions on the personality traits of TMTs on the upper echelons theory, ignoring the pressure of external stakeholders on TMTs and the strategic adjustment of executives. In this study, the new variable of TMT media exposure is introduced into the research of the upper echelons theory. By studying its influence on CSR strategic decision-making, the study of external stakeholders’ pressure on TMTs is promoted along with further development of the upper echelons theory and the stakeholder theory. At the same time, the external stakeholders in the research field, such as media exposure (Ramadhini et al., 2020), are expanded.

Secondly, TMT power and TMT political connection are introduced into the research of TMT media exposure and CSR as moderating variables from the perspective of TMT characteristics (upper echelons theory). We find that TMT power and TMT political connection play a negative role in regulating the relationship between TMT media exposure and CSR, which is contrary to most previous views, and supplements the rare literature on the negative influence of TMT power (Jiraporn and Chintrakarn, 2013; Velte, 2019) and TMT political association (Huang and Zhao, 2016).

Thirdly, it broadens the research perspective of CSR. CSR plays an important role in promoting the progress of enterprises, which has been generally recognised by society and academia. Many achievements can be referenced by academia for the influencing factors of corporate social responsibility, but these achievements are basically at the company level, such as corporate governance. As for the influencing factors of CSR, many academic achievements can be referred to, but these achievements are basically at the firm level, such as corporate governance. There is little research on internal managers, such as senior management and CEO.

Since Hambrick and Mason (1984) put forward the concept of the upper echelons theory, the combination of TMT characteristics and CSR has gradually become a research hotspot in academic circles. A large number of relevant research results and literature have sprung up, and relevant conclusions have been drawn. At the same time, the proponent of the upper echelons theory has been calling on management scholars to learn from the research results of psychology, sociology, and communication and introduce new variables into the research of TMT characteristics. Media attention is an important research variable in the field of communication. However, this important characteristic has not been paid attention to by the field of management, especially the field of strategic management. In this study, the important variable of TMT media exposure is introduced into the research of CSR, and it is found that TMT media exposure exerts pressure on TMTs (Wallack and Dorfman, 1996), increasing administrative intervention, and influences corporate strategic decisions. In a word, the introduction of TMT media exposure, a new research variable, has greatly enriched and expanded the research on CSR.

#### Practical Implications

This research also has important practical implications. Firstly, it gives full play to the media’s function of supervising TMTs, which compensates for that which cannot be covered by the existing legal system. TMT media attention emphasis on CSR supervision cannot be separated from the timely and objective exposure of media. The government should strengthen legal guarantees and economic support for the media, improve the enthusiasm, impartiality, and credibility of news media exposure, exert pressure on enterprises through the non-legal system of the media, and promote CSR practices. At the same time, media attention to executives is a double-edged sword, bringing public opinion pressure not only to TMTs but also to companies as a whole. If TMTs handle this attention properly, it is easier to arouse investors’ confidence in the capital market and safeguard the public’s interests and vice versa.

Secondly, powerful TMTs reduce TMT media attention on CSR fulfilment, which shows that the agency problem caused by powerful TMTs is still the primary problem in the process of corporate governance in China. Under the background that the public and investors are paying more and more attention to the corporate sustainable development level, media exposure can effectively promote the managers of listed companies to pay attention to social responsibilities. Corporate controllers should balance the self-interest managerial behaviours in the decision-making process through reasonable incentive mechanisms. At the same time, they should constantly improve the corporate internal control level and form effective supervision and restraint for strong managers to avoid abuse of power.

Thirdly, the present article supplements the sparse literature on the benefits of a lack of political connections. “Rule No. 18,” issued by the Communist Party of China on October 19, 2013 mandates that no government or party officials, including both incumbent officials and those who have resigned or retired within the past 3 years, should serve on boards of directors or receive payments from private firms (Wei et al., 2020). This study provides further elaboration of this policy. It shows that building a healthy relationship between government and business and allowing listed companies to fully compete in the marketplace facilitates them to actively fulfil their CSR through stakeholder pressure.

### Limitations and Future Research Directions

This study has some limitations. Its predecessors only used print media as a source of data on media exposure, while the present study adds network media as a source of media reports. However, with the popularity of Weibo, Tiktok, WeChat, Twitter, and other internet-based media communication platforms, whether TMT exposure affects CSR information disclosure still requires further study. Secondly, the research in media reports is often cross-sectional and focuses on a certain level of analysis. Thus, it is hoped that in the future research literature, the institutional, organisational (Garcia-Sanchez et al., 2014), and individual levels can be combined in multi-level and longitudinal studies. Finally, this article does not discuss whether external media governance mechanisms and internal corporate governance mechanisms are complementary or substitutes for each other in the fulfilment of CSR nor does it discuss the role of executive media coverage in the governance of CSR in different external contexts. Further investigation may be needed in the future.

We acknowledge the invaluable input by one of our reviewers who directed our attention to the following challenges. (1) The relationship between the media outlets and corporate financing and advertising expenses. (2) Because the journalists are afraid of being sued by the company, there is a certain deviation in the reported content which cannot better reveal the internal corporate governance information. (3) Media could be used in political conflicts or debates (manipulation of the media by political competitors of the TMT). The reports of political conflicts by mass media often have a strong ideology and certain national tendency so as to affect the influence of the audience and strive for the initiative of public opinion. Therefore, political conflict has always been the focus of social mass media. As the embodiment of justice, the guiding function of mass media directly affects the choice and orientation of the public. (4) There is a necessary difference between advertising in the media and critical objective analysis. In short, the Chinese media also pay strong attention to the material conflict between the group and the government, which means that the coal body no longer regards the conflict between the officials and the people as a restricted area. It blindly maintains a high degree of consistency with the ideology of the party and the government, but objectively reflects the problems existing in the social reality and criticises and supervises the government power, which is great progress in the supervision of public opinion by the Chinese media.

## Data Availability Statement

Publicly available datasets were analysed in this study. This data can be found here: http://www.rksratings.cn.

## Author Contributions

YJ conducted the statistical analysis. LZ and YJ conducted the data analysis. LZ contributed to all the phases of the study from conception and design of the study, results interpretation, and writing manuscript and also mainly responsible for replying to the comments of reviewers. HT was responsible for the proofreading of the manuscript. All the authors agreed to all aspects of the work, and approved the version to be published.

## Conflict of Interest

The authors declare that the research was conducted in the absence of any commercial or financial relationships that could be construed as a potential conflict of interest.

## Publisher’s Note

All claims expressed in this article are solely those of the authors and do not necessarily represent those of their affiliated organizations, or those of the publisher, the editors and the reviewers. Any product that may be evaluated in this article, or claim that may be made by its manufacturer, is not guaranteed or endorsed by the publisher.

## Footnotes

1
http://www.rksratings.cn/
